# Testing the Effectiveness of an International Conservation Agreement: Marketplace Forensics and CITES Caviar Trade Regulation

**DOI:** 10.1371/journal.pone.0040907

**Published:** 2012-07-25

**Authors:** Phaedra Doukakis, Ellen K. Pikitch, Anna Rothschild, Rob DeSalle, George Amato, Sergios-Orestis Kolokotronis

**Affiliations:** 1 Scripps Institution of Oceanography, University of California San Diego, La Jolla, California, United States of America; 2 Institute for Ocean Conservation Science, Stony Brook University, Stony Brook, New York, United States of America; 3 Sackler Institute for Comparative Genomics, American Museum of Natural History, New York, New York, United States of America; 4 Department of Biology, Barnard College, Columbia University, New York, New York, United States of America; Biodiversity Insitute of Ontario - University of Guelph, Canada

## Abstract

**Background:**

The international wildlife trade is a key threat to biodiversity. Temporal genetic marketplace monitoring can determine if wildlife trade regulation efforts such as the Convention on International Trade in Endangered Species (CITES) are succeeding. Protected under CITES effective 1997, sturgeons and paddlefishes, the producers of black caviar, are flagship CITES species.

**Methodology/Principal Findings:**

We test whether CITES has limited the amount of fraudulent black caviar reaching the marketplace. Using mitochondrial DNA-based methods, we compare mislabeling in caviar and meat purchased in the New York City area pre and post CITES listing. Our recent sampling of this market reveals a decrease in mislabeled caviar (2006–2008; 10%; *n* = 90) compared to pre-CITES implementation (1995–1996; 19%; *n* = 95). Mislabeled caviar was found only in online purchase (*n* = 49 online/41 retail).

**Conclusions/Significance:**

Stricter controls on importing and exporting as per CITES policies may be having a positive conservation effect by limiting the amount of fraudulent caviar reaching the marketplace. Sturgeons and paddlefishes remain a conservation priority, however, due to continued overfishing and habitat degradation. Other marine and aquatic species stand to benefit from the international trade regulation that can result from CITES listing.

## Introduction

The lucrative international trade in wildlife is a key threat to global biodiversity [1]–[3]. As species threatened by trade may also face overexploitation for domestic use and habitat degradation, the impact of international wildlife trade policy must consider more than trends in abundance or species status [4]. Trade policy effectiveness can be tracked through endpoint market monitoring, which can be particularly useful in detecting illegally traded products [5].

Genetic applications facilitate identification of the species origin of marketplace products to determine if illegal products are present [5]. If done in real time, exploitation can also be curtailed in geographic areas where illegal harvest is occurring [6]. Genetic marketplace monitoring incorporating a temporal component [7] permits detection of changes in species presence over time thereby demonstrating policy effectiveness and dictating possible policy change.

The Convention on International Trade in Endangered Species (CITES) is the most important international wildlife trade treaty [2]. The treaties measurable outcomes include eliminating trade in highly endangered species (Appendix I), allowing controlled trade for at-risk species that could benefit from the revenue and conservation incentive of sustainable trade (Appendix II), and sometimes cultivating trade in captive-origin materials [8]–[9]. Marine and aquatic species are increasingly targeted for CITES listing given that many species are in decline, international trade regulation could complement fisheries management, and seafood mislabeling is on the rise [10]–[13]. Demonstrating that CITES has resulted in conservation benefit for listed marine and aquatic species could help facilitate listing efforts for unlisted species.

**Table 1:** Samples tested in the present caviar species identification survey.

Labeled species	In-store		Internet		Price Range	GenBank accession nos. (sample number in parentheses)	
	2006	2008	2006	2008	(per oz US $)	*Cytb* or *coxI*	D-loop
Beluga(*Huso huso*)	1, 13		23, 30*,32, 38*	57	114.00–195.00	JX238370(1), JX238369(13), JX238424(23),JX238402(30), JX238425(32), JX238387(38),JX238368(57),	JX213602(30), JX213607(38)
Sevruga(*Acipenser stellatus*)	3, 6, 9,12, 18	56, 58,71, 92	25, 28,31, 33*,36*	60, 64*,69, 75*,76, 77,84	60.00–161.00	JX238379(3), JX238430(6) JX238380(9),JX238381(12), JX238426(18), JX238429(25),JX238427(28), JX238428(31), JX238350(33),JX238431(36a), JX238408(36b), JX238375(56),JX238373(58), JX238372(60),JX238383-4(64a–b), JX238376(69),JX238374(71), JX238349(75a), JX238352(75b),JX238377(76), JX238371(77), JX238382(84),JX238378(92)	JX213605-6(36a–b)
Ostera(*A. baerii*)	2(F), 8(F)	51(F),54(F),85(F)	24(F)	81*(F),94 (F)	20.00–85.00	JX238385(2), JX238386(8), JX238422(24),JX238389(51), JX238391(54), JX238398(81),JX238394(85)	JX213594(2), JX213595(8), JX213599(24), JX213609(51), JX213611(54), JX213620(81), JX213623(85)
Ostera(*A. gueldenstaedtii*)			27	68, 82(F),83, 86(F),87, 93*(F)	45.00–175.00	JX238432(27), JX238405(68), JX238399(82),JX238400(83), JX238406(86), JX238407(87),JX238395(93),	JX213600(27), JX213617(68), JX213621-22(82–83), JX213624-25(86–87), JX213626(93)
Osetra(*A. gueldenstaedii*x *A. baerii*)	14(F)				85.00	JX238409(14)	JX213597(14)
Osetra(*A. persicus*)	55		29	63, 73	119.00–150.00	JX238433(29), JX238403(55), JX238411(63),JX238397(73)	JX213601(29), JX213612(55), JX213614(63), JX213619(73)
Osetra(unspecified)	10, 20(F),21	44, 47(F),48(F),53(F),70	34, 35(F)	62, 65(F),67	60.50–220.00	JX238404(10), JX238413(20), JX238434(21),JX238435(34), JX238423(35), JX238410(44),JX238418(47), JX238388(48), JX238390(53),JX238392(62), JX238393(65), JX238401(67),JX238396(70)	JX213596(10), JX213598(21), JX213603-4(34–35), JX213608(48), JX213610(53), JX213613(62), JX213615(65), JX213616(67), JX213618(70)
White(*A. transmontanus*)	11(F), 15	45(F),52(F, M),90 (F)	40(F)		32.50–75.00	JX238412(11), JX238415(15), JX238416(40),JX238414(45), JX238418(52), JX238421(90)	
Paddlefish(*Polyodon spathula*)	4, 16,19, 22	43, 50	26	61, 66,72, 80,89	16.50–57.00	JX238437(4), JX238436(16), JX238346(19),JX238438(22), JX238358(26), JX238357(43),JX238356(50), JX238355(61), JX238354(66),JX238353(72), JX238347(80), JX238351(89)	
Hackleback(*Scaphirhynchus platorynchus*)	5, 7, 17	49	39	74, 79,91	15.00–28.00	JX238359(5), JX238365(7), JX238360(17),JX238366(39), JX238367(49), JX238362(74),JX238363(79), JX238364(91)	
American-unspecified		46	41(F)	59, 88(F)	48.00–75.00	JX238417(41), JX238348(46), JX238361(59),JX238420(88)	
CaviarSubstitute				95	15.00	N/A	N/A
Caspian SeaBlack Caviar				96*	7.00	JX238439(96-*cox*1 gene)	
Total	23	19	18	33			

* mislabeled caviar lot (see Table 2 for identification), F: labeled as “farmed”, M: meat sample.

**Figure 1:**
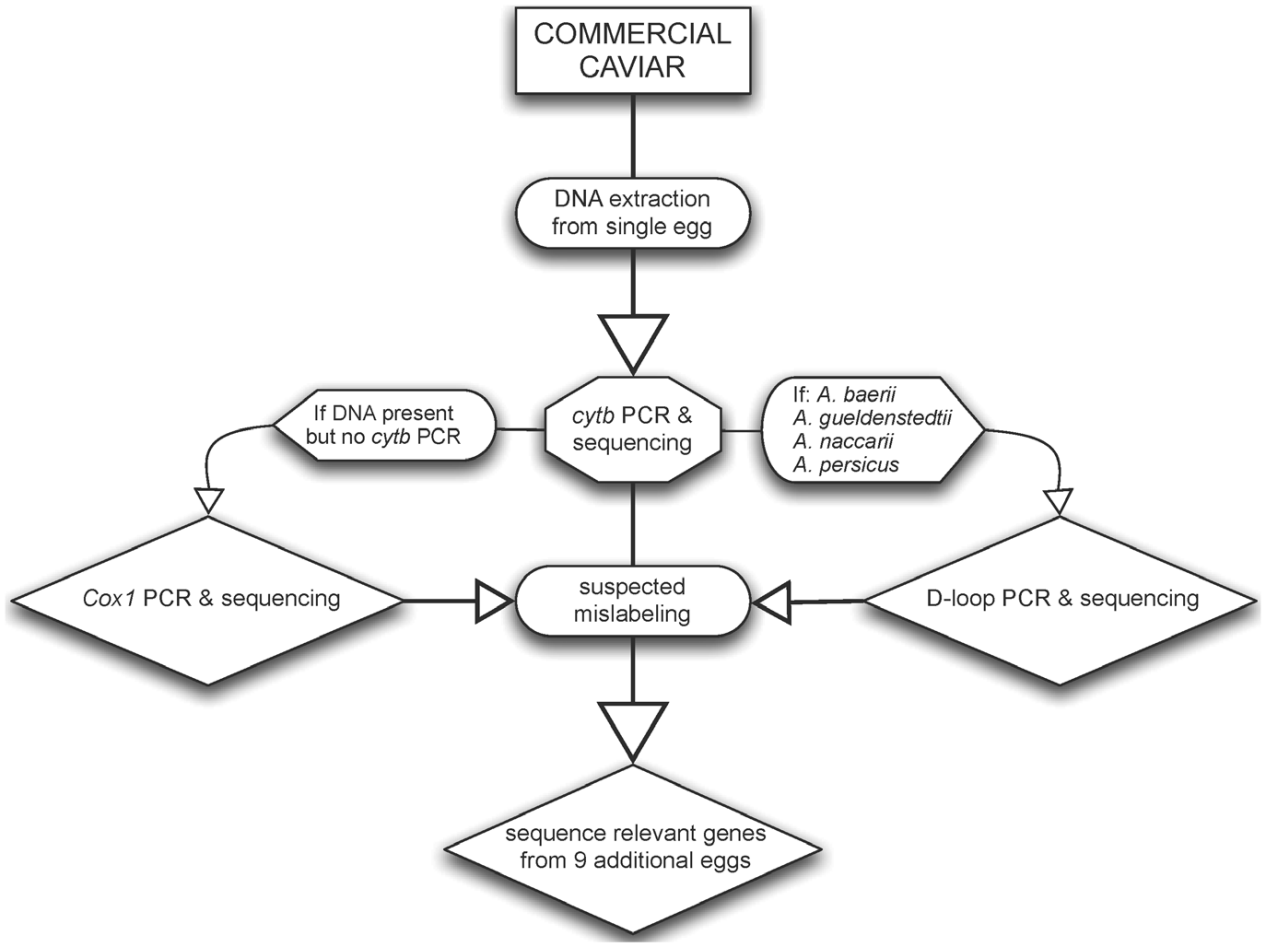
Workflow for DNA-based method for caviar species identification.

Sturgeons and paddlefishes (Order Acipenseriformes), the producers of black caviar, are flagship CITES species. The group has been listed under CITES since 1997 due to widespread population abundance declines from overfishing and habitat degradation and illegal trade in caviar [14]. Most species are listed under Appendix II [8], [14]. The 25 species of sturgeon and two species of paddlefish inhabit the Northern hemisphere and are mostly anadromous, with all reproducing in freshwater. As serial overfishing affected the productivity of sturgeon fisheries, the black caviar harvest historically shifted from eastern North America and Western Europe to the Black and Caspian Sea. The black caviar trade is a valuable global industry with aquaculture production beginning to surpass wild [14]–[15]. Species in the genus *Acipenser*, *Huso*, *Polyodon* and *Scaphirhynchus* are commercially exploited. Phylogenetic studies find some genera polyphyletic and species complexes hard to distinguish using mitochondrial DNA (mtDNA) [16], while nuclear DNA approaches have been limited by the polyploidy characteristic of the group.

**Figure 2:**
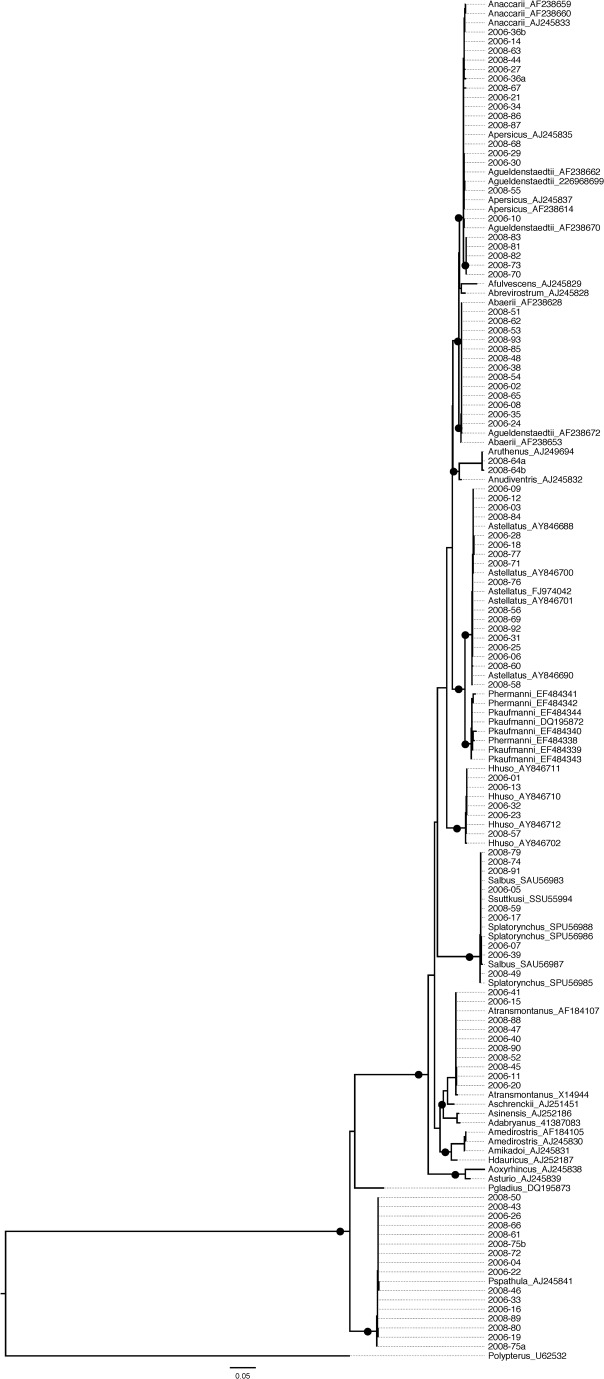
Best maximum likelihood phylogenetic tree of all *cytb* sequences. Sequences from this study are coded by the year of sampling and the sample number. GenBank sequences are indicated by their corresponding accession numbers. Black circles denote well-supported nodes (>90%). LnLik = –4882.55. *α* = 0.223717.

As the species origin of caviar is difficult to determine by visual inspection and illegal trade and mislabeling is problematic, molecular methods were developed to determine species origin [17]–[20]. Reviews highlighting these methods illustrate drawbacks and the need for multi-gene approaches [21]–[22]. At present, customs agents in Europe, the USA, and Canada genetically test random samples of caviar imports for species authenticity using mtDNA techniques. These methods fail to detect hybrids, which are rare in the wild, but increasing with environmental perturbations (e.g. river dams) and non-native species release and are also common in aquaculture [23]. Population-assignment markers are also presently not useful at a taxonomic scale applicable for screening caviar.

**Figure 3:**
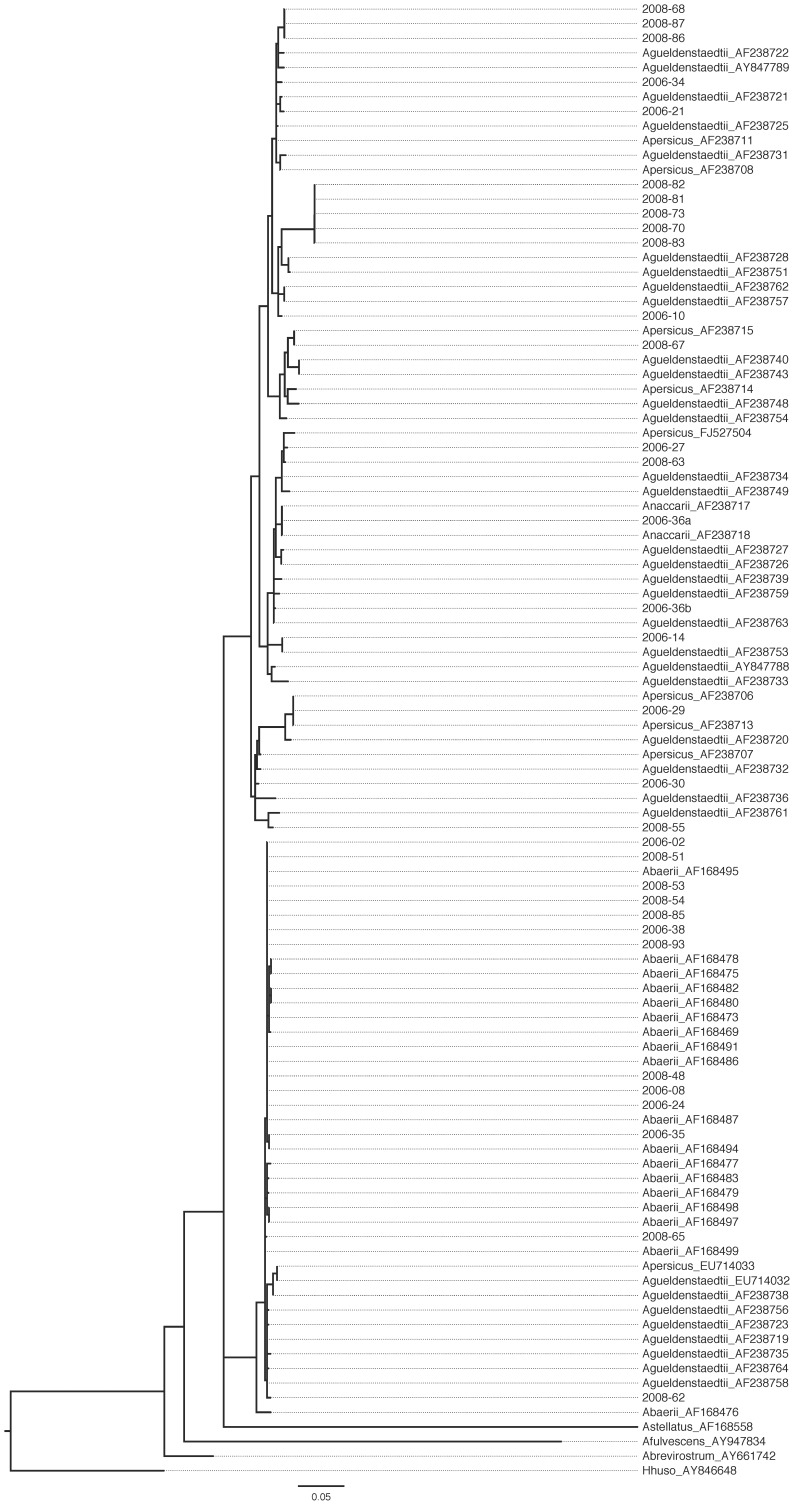
Best maximum likelihood phylogenetic tree of all D-loop sequences from this study. Sequences from this study are coded by the year of sampling and the sample number. GenBank sequences are indicated by their corresponding accession numbers. LnLik = –3121.95. *α* = 0.345333.

We sought to use the most widely applied mtDNA species identification techniques for black caviar to examine the New York City market pre and post CITES listing to gauge whether CITES policies have reduced or eliminated fraudulent products in the marketplace and detect if the nature of illegal trade has changed. To do this, we repeat a previous market survey (1995–1996) that revealed that 19% of commercially available caviar in the New York City area was mislabeled with respect to species origin [17]–[18], [24]. By sampling the same market more than 10 years later (2006–2008), we examine whether CITES implementation has resulted in conservation improvements in one major market.

**Table 2:** Mislabeled samples as identified through *cytb*, D-loop and/or *cox1* sequencing.

Lot #	Labeled species	*n*	*n* H	MtDNA identification
30	Beluga	14	1	*Acipenser gueldenstaedtii*/*A. naccarii* (*cytb*, D-loop)
33	Sevruga (Caspian/Black Sea)	14	1	*Polyodon spathula* (*cytb*)
36	Sevruga (Caspian Sea)	10	2	*A. gueldenstaedtii*/*A. naccarii* (*cytb*, D-loop)
38	Beluga (Caspian Sea)	10	1	*A. baerii* (*cytb*, D-loop)
64	Sevruga (Kazakhstan)	10	2	*A. ruthenus* (*cytb*)
75	Sevruga	10	2	*P. spathula* (*cytb*)
81	Osetra (*A. baerii*)	10	1	*A. gueldenstaedtii*/*A. naccarii* (*cytb*, D-loop)
93	Osetra (*A. gueldenstaedtii*)	10	1	*A. baerii* (*cytb*, D-loop)
96	Caspian Sea Black Caviar	11	2	*Esox lucius* (*cox1*)

*n*: number of eggs sampled, *n* H: number of haplotypes detected.

## Results

DNA was successfully extracted from 90 caviar lots and one meat sample, with different genes explored to accomplish species identification (Table 1, Figure 1). Sequences for the *cytb* gene region were obtained for the meat sample and 89 of the 92 caviar lots (Figure 2). The control region (D-loop) was sequenced for samples from 32 lots, including “osetra” (*n* = 29) samples plus three samples with *cytb* sequences corresponding to *A. baerii*, *A. gueldenstaedtii*, *A. naccarii*, or *A. persicus* (Figure 3). Two “osetra” samples (#44, *#*94) did not PCR amplify for the D-loop locus. Control region sequencing permitted assignment as *A. baerii*, *A. baerii*-like *A*. *gueldenstaedtii*, or *A. gueldenstaedtii*/*A. naccarii* (Figure 3). Assignment to *A. persicus* was not possible consistent with previous studies (Figure 3) [18].

DNA could not be extracted from two samples (#94 “*A. baerii*”, #95 “Caviar Substitute”). Total DNA electrophoresis and spectrophotometer analysis indicated no evidence of DNA. For sample #96, *cytb* and D-loop primers failed and the *cox1* region was sequenced.

Mislabeling was detected in nine lots of caviar (see below) so multiple sequences were examined (Table 2). In three cases (#36, #64, #75) different haplotypes were recovered (Figures 2 & 3) but species identification was unaffected. The *cytb* sequencing permitted identification of two samples of *Polyodon spathula* labeled as sevruga (#33, #75) and one sample of *A. ruthenus* labeled as sevruga (#64) (Table 2). For the sample that required *cox1* sequencing (#96 labeled as “black caviar from the Caspian Sea”), testing of 11 samples indicated a match Northern Pike (*Esox lucius*) in a GenBank MegaBLAST search (E-value = 0.0, pairwise identity = 98%–100%, accession no. FJ890069.1). The D-loop locus confirmed mislabeling in five cases (#30, #36, #38, #81, #93; Table 2). The mislabeled products were all purchased on the Internet (2006: #30, #33, #36, #38; 2008: #64, #75, #81, #93, #96; Tables 1 & 2).

## Discussion

The level of mislabeling detected (10%) is almost half that found in the previous study (19%) [17]–[18], [24]. We found no cases of fraud in samples from retail shops as compared to numerous cases previously [24]. The decrease in fraud could be due to the tighter controls over international trade and import as implemented through CITES and US agencies and random genetic testing performed by US officials upon import. There may also be a perception among retail establishments of greater scrutiny.

Some differences emerge in comparing the kind of mislabeling found here to that discovered previously. We did not find ship sturgeon (*A. nudiventris*) as a replacement, which is encouraging given it is Critically Endangered [25]. Similarly, no instances of substitution with *A. schrenckii* or *A. transmontanus* were detected. In the previous study, caviar labeled as “American sturgeon” was mislabeled in multiple instances. Finding no such cases here suggests that US labeled products may now be better regulated. Use of *A. ruthenus*, *A. baerii* (likely farm raised), and Northern Pike were unique to the present study (Table 2). Finding multiple haplotypes in individual caviar tins did not occur previously, suggesting that caviar from multiple individuals may now be pooled.

The motivation for substitution likely varied. Replacement of beluga with *A. gueldenstaedtii, A. naccarii,* or *A. baerii* (#30, #38) could be due to the scarcity of beluga sturgeon or the 2004 Endangered Species Act listing of beluga sturgeon that made US import of beluga illegal as of 2005. If this caviar (purchased in 2006) had been confirmed as beluga, it could have been legal given caviars’ shelf-life of 18 months. Purchased beluga caviar was not labeled with the year of harvest with one exception: a 2006 sample (#1) was labeled as from the 2006 harvest and thus was likely illegal. The single beluga caviar sample purchased in 2008 was not labeled and was either close to three years old or illegally imported. Similarly, substitution of *A. ruthenus* for sevruga (#64) represents a case of illegal import as well as mislabeling. *Acipenser ruthenus* CITES export quotas have not been issued since 2005. While *A. stellatus* and *A. ruthenus* may overlap in distribution, they are easily visually identified.

Increased profit fueled replacement in other instances, including substituting sevruga with *P. spathula* (#33, #75; Table 1, 2). This is also true for the Northern Pike sample (#96) that was advertised as a product of the Caspian Sea. The two cases of using *P. spathula* as an illegal replacement indicate that North American species may be under increased pressure as Caspian Sea species decline [26]. The caviar of *A. stellatus* and *P. spathula* are similar in size, easing substitution.

In two cases, *A. baerii* and *A. gueldenstaedtii* were substituted for one another (#81, #93). These species do not overlap in geographic distribution, with *A. baerii* inhabiting lakes and rivers of Siberia and *A. gueldenstaedtii* found in the Caspian and Black Sea regions. Introgression between the species occurs in the Caspian and Azov Sea where animals morphologically identified as *A. gueldenstaedtii* have mtDNA haplotypes similar to *A. baerii*, potentially as a result of release from aquaculture [18], [27]. Distinguishing between *A. baerii* and *A. baerii-like gueldenstaedtii* can be accomplished through the D-loop sequence-based method applied here [20]. Sample #81 was labeled as farmed *A. baerii* but our testing indicates *A. gueldenstaedtii*. This could be an instance where wild product is being falsely sold as a product from aquaculture. Sample #93 was labeled as wild Caspian Sea *A. gueldenstaedtii* caviar while it tested as *A. baerii*. Either [20] did not detect all variation present in the Caspian Sea population or the sample is mislabeled. If it is mislabeled, it could represent farmed *A. baerii* being used as a replacement for wild *A. gueldenstaedttii* caviar since little wild *A. baerii* harvest occurs. Overall, the illegal use of *A. gueldenstaedtii*, as well as *A. ruthenus*, and *P. spathula* is cause for conservation concern. Better tracking of international trade may be needed in the case of the first two species while national controls are needed for all three species.

Although we use only two mitochondrial genes for our forensic identification, our study pinpointed the maternal species identity of commercially available caviar in most cases. An exception was for species in the genus *Scaphirhynchus*, which are difficult to identify using mtDNA due to marker sensitivity and hybridization [28]. We assumed these samples were legally harvested *S. platorhynchus*. Additional research is needed to confirm the species integrity of *A. persicus*, as suggested previously [18], [29]. Improvements to the overall method would include incorporating markers to identify hybrid-origin caviar, detect population origin [30], and differentiate farmed from wild caviar [31]–[32].The latter will be especially helpful as aquaculture production increases [15], [32].

Our temporal analysis indicates that CITES trade regulation is having a positive effect, confirming the utility of the most important international treaty regulating the trade in wildlife for a group of globally traded aquatic and marine species. While some species appear to be less susceptible to illegal sale in the US market, others are now more vulnerable. Unfortunately, the conservation status of many sturgeon species has not improved since CITES listing, with most commercially traded species now Critically Endangered [33] and many, such as beluga, currently being overfished [34]. Limiting fishing in range states and further curtailing trade will be necessary to allow depleted sturgeon populations to recover.

Recent debate over the utility of CITES listing for marine and aquatic species has included whether CITES is an appropriate instrument for species managed by fisheries agencies and other international agreements. Our study suggests that listing can positively impact international trade controls for such taxa. Fisheries management would need to complement such efforts, however, for conservation to be achieved.

## Materials and Methods

### Sampling

Ninety-two (92) tins of caviar and one (1) piece of sturgeon meat were purchased for study in 2006 (*n* = 41) and 2008 (*n* = 52) (Table 1). Forty-two samples were acquired from nine gourmet shops in the New York City area and 51 online from 12 web retailers in New York, New Jersey, and Florida as well as E-Bay (Table 1). We included the same shops and species distribution as sampled previously (14 shops *n* = 79, 7 web retailers *n* = 26) [35] with some exceptions. Nine retailers no longer sold caviar and certain caviars were available only in limited supply (e.g., beluga, sevruga). Overall, our approach represents a random sample of the caviar available on the New York City market as in our original study.

### Laboratory Procedures

All information present on the product label, including the purported species origin was noted from each tin. DNA from one egg per tin was extracted using a DNeasy Blood and Tissue Kit (Qiagen). A single egg was studied since mixing was not detected previously [24]. Additional eggs were sampled when mislabeling was detected; see below. DNA from a small (25 mg) sample of the sturgeon meat was extracted using the same method.

A portion of the *cytochrome b* (*cytb*) gene region was sequenced for each sample. For most, PCR amplification used primers B1 (5′-CCATCCAACATCTCTGCTTGA TGAAA-3′) and S2A (5′- AGTACTCACATGAATTGGAGG-3′) [18] and the following protocol: 1.25 U *Taq* DNA polymerase (Fisher Scientific), 2.5 µL 10× Buffer A, 2.5 µL 8 mM dNTPs, 0.5 µL 250 mg/mL BSA, 1.25 µL of each 10× primer, 15.75 µL ddH_2_O, and 1 µL of DNA sample. Thermal conditions for PCR were 94°C for 3 min followed by 8 cycles of 94°C for 1 min, 49°C for 1 min, 72°C for 110 s, followed by 30 cycles of 94°C for 1 min, 47°C for 1 min, 72°C for 110 s, with a final extension of 72°C for 10 min. We also employed Illustra puReTaq Ready-To-Go PCR beads (GE Healthcare), 21 µL ddH_2_O, 1 µL each 10× primer, and 1 µL of DNA sample when the first protocol failed. Thermal conditions for this PCR were 94°C for 3 min followed by 8 cycles of 94°C for 1 min, 53°C for 1 min, 72°C for 110 s, followed by 30 cycles of 94°C for 1 min, 51°C for 1 min, 72°C for 110 s, with a final extension of 72°C for 10 min. Samples that failed to amplify under either protocol were cleaned with a QIAQuick PCR Purification Kit (Qiagen) using 50 µL of the original DNA extraction and re-amplified.

Samples labeled as hackleback sturgeon (*Scaphirhynchus platorynchus)* did not amplify with the standard protocol. PCR was instead performed using H15915-stur (5′- CCTTCGATCTTCGGTTTACAAGAC-3′) and L14724 (5′- GTGACTTGAAAAACCACCGTTG-3′) [28] and the following protocol: 14.75 µL ddH_2_O, 1.25 U AmpliTaq Gold (Applied Biosystems, Inc. (ABI)), 2.5 µL 10× Gold Buffer, 2.5 µL 8 mM dNTPs, 1.5 µL 25 mM MgCl_2,_ 0.5 µL 250 mg/mL BSA, 1.25 µL of each 10× primer, 14.25 µL ddH_2_O, 1 µL of sample. Thermal conditions were 94°C for 2 min, followed by 35 cycles of 94°C for 1 min, 55°C for 1 min, 72°C for 90 s, with a final extension of 72°C for 10 min.

All sequencing used the BigDye v3.1 chemistry kit (ABI) on an ABI 3730×l DNA Analyzer. When the analysis indicated mislabeling (see below), at least nine more eggs were extracted and sequenced for *cytb* to confirm mislabeling and detect mixing. After identifying a sample as *Acipenser baerii, A. gueldenstaedtii, A. naccarii, or A. persicus* with *cytb*, the sample was sequenced for the D-loop to increase species identification resolution (see [18], [20]). Most D-loop PCR reactions used primers dlp1.5 (5′- GCACCCAAAGCTGARRTTCTA-3′) and H00651 (5′- ATCTTAACATCTTCAGTG-3′) [18] and the following protocol: 1 U *Taq* DNA polymerase (Fisher Scientific) 2.5 µL 10× Buffer A, 2.5 µL 8 mM dNTPs, 1.5 µL 250 mg/mL BSA, 1 µL of each 10× primer, 15.3 µL ddH_2_O, and 1 µL of sample. Thermal conditions were 94°C for 3 min followed by 33 cycles of 94°C for 1 min, 46°C for 1 min, and 72°C for 90 s, with a final extension time of 10 min. The sequencing primer AHR3 (5′- CATACCATAATGTTTCATCTACC-3′) [18] replaced dlp1.5 in sequencing reactions. Samples that did not amplify with dlp1.5 and H00651 were amplified using primers L16615 (5′- CACCCTTAACTCCCAAAGCTAAGATTC-3′) and H1144 (5′- CCTCACAGGAATGCGGAGACTTGC-3′) with the following protocol: 1.25 U *Taq* DNA polymerase (Fisher Scientific) 2.5 µL 10× Buffer A, 2.5 µL 8 mM dNTPs, 0.5 µL 250 mg/mL BSA, 1.25 µL of each 10× primer, 15.75 µL ddH_2_O, and 1 µL of sample. Thermal conditions were 94°C for 3 min followed by 33 cycles of 94°C for 1 min, 59°C for 1 min, and 72°C for 90 s, with a final extension time of 10 min. These products were sequenced with primers L16615 and H1144, along with two additional sequencing primers: L195 (5′- TGTAGTAAGAGCCGAACAT-3′) and H905 (5′- TCGATGACAAGTCAGTCCTG-3′). In cases where *cytb* and D-loop primers failed, a portion of the cytochrome *c* oxidase subunit I (*cox1*) gene was amplified using conditions in [36]. The overall protocol flowchart is detailed in Figure 1.

### Data Analysis

Initial species identifications were made using the nucleotide MegaBLAST [36] in the NCBI GenBank database (http://www.ncbi.nlm.nih.gov/genbank). Species identifications were confirmed in a phylogenetic tree context using maximum likelihood (ML) as an optimality criterion. Tree searches were implemented in the fine-grained parallel Pthreads (POSIX Threads Library) build of RAxML v7.3.0 [38]–[39] using the general time-reversible nucleotide substitution model [40]–[41] with among-site rate heterogeneity modeled by the Γ distribution and four rate categories [42] (*α*_cytb_ = 0.223717; *α*_D-loop_ = 0.345333). Ten independent tree searches were run based each on a stepwise-addition maximum parsimony starting tree. Outgroups included a *Huso huso* D-loop sequence (GenBank accession no. AY846648) and a *Polypterus ornatipinnis cytb* sequence (U62532) based on known phylogenies. Candidate reference nucleotide D-loop and *cytb* sequences were downloaded from GenBank (see accession numbers in Figures 2 & 3). For samples analyzed for the D-loop, sequences were checked against the primer sequences designed by [20]. Node robustness on the trees was estimated using 500 rapid bootstrap pseudoreplicates [43]. The best ML tree for each dataset was filtered through the swarm of bootstrap trees and node support values reflect the proportion of bootstrap tree nodes in agreement with the nodes of the best ML tree.

## References

[1] Broad S, Mulliken T, Roe D, The trade in Wildlife: Regulation for Conservation. (Oldfield S (2003). The nature and extent of legal and illegal trade in wildlife..

[2] Phelps J, Webb EL, Bickford D, Nijman V, Sodhi NS (2010). Boosting CITES.. Science.

[3] Rosen GE, Smith KF (2010). Summarizing the evidence on the international trade in illegal wildlife.. EcoHealth.

[4] Donald PF, Sanderson FJ, Burfield IJ, Bierman SM, Gregory RD (2007). International conservation policy delivers benefits for birds in Europe.. Science.

[5] Baker CS (2008). A truer measure of the market: the molecular ecology of fisheries and wildlife trade.. Mol Ecol.

[6] Wasser SK, Clark WJ, Drori O, Kisamo ES, Mailand C (2008). Combating the illegal trade in African Elephant ivory with DNA forensics.. Conserv Biol.

[7] Schwartz MS, Luikart G, Waples RS (2007). Genetic monitoring as a promising tool for conservation and management.. Trends Ecol Evol.

[8] Ginsberg J (2002). CITES at 30, or 40.. Conserv Biol.

[9] Uscamaita MR, Bodmer R (2010). Recovery of the Endangered giant otter *Pteronura brasiliensis* on the Yavarí-Mirín and Yavarí Rivers: a success story for CITES.. Oryx.

[10] Ogden R (2008). Fisheries forensics: the use of DNA tools for improving compliance, traceability and enforcement in the fishing industry.. Fish Fish.

[11] Jacquet JL, Pauly D (2008). Trade secrets: Renaming and mislabeling of seafood.. Mar Pol.

[12] Doukakis P, Parsons ECM, Burns WCG, Salomon AK, Hines E (2009). Gaining traction: Re-treading the wheels of marine conservation.. Conserv Biol.

[13] Stokstad E (2010). Trade trumps science for marine species at international meeting Science.

[14] Pikitch EK, Doukakis P, Lauck L, Chakrabarty P, Erickson DL (2005). Status, trends and management of sturgeon and paddlefish fisheries.. Fish Fish.

[15] Bronzi P, Rosenthal H, Gessner J (2011). Global sturgeon aquaculture production: an overview.. J Appl Ichthyol.

[16] Krieger J, Hett AK, Fuerst PA, Artyukhin E, Ludwig A (2008). The molecular phylogeny of the order Acipenseriformes revisited.. J Appl Ichthyol.

[17] DeSalle R, Birstein VJ (1996). PCR identification of black caviar.. Nature.

[18] Birstein VJ, Doukakis P, DeSalle R (2000). Polyphyly of mtDNA lineages in the Russian sturgeon, *Acipenser gueldenstaedtii*: forensic and evolutionary implications.. Conserv Genet.

[19] Congiu L, Dupanloup I, Patarnello T, Fontana F, Rossi R (2001). Identification of interspecific hybrids by amplified fragment length polymorphism: the case of sturgeon.. Mol Ecol.

[20] Mugue NS, Barmintseva AE, Rastorguev SM, Mugue VN, Barmintsev VA (2008). Polymorphism of the mitochondrial DNA control region in eight sturgeon species and development of a system for DNA-based species identification.. Russ J Genet.

[21] Ludwig A (2006). A sturgeon view on conservation genetics.. Eur J Wildl Res.

[22] Ludwig A (2008). Identification of Acipenseriformes species in trade.. J Appl Ichthyol.

[23] Ludwig A, Lippold S, Debus L, Reinartz R (2009). First evidence of hybridization between endangered sterlets (*Acipenser ruthenus*) and exotic Siberian sturgeons (*Acipenser baerii*) in the Danube River.. Biol Inv.

[24] Birstein VJ, Doukakis P, Sorkin B, DeSalle R (1998). Population aggregation analysis of caviar producing species of sturgeon and implications for diagnosis of black caviar.. Conserv Biol.

[25] Kottelat M, Gesner J, Freyhof J (2009). *Acipenser nudiventris*. IUCN 2010 Red List of Threatened Species. Version 2010.4.. http://www.iucnredlist.org.

[26] Bettoli PW, Kerns JA, Scholten GD, Paddlefish Management, Propagation, Conservation in the 21st Century: Building from 20 Years of Research, Management. (Paukert CP, Scholten GD (2009). Status of paddlefish in the United States.. ) Am Fish Soc Symp Ser.

[27] Jenneckens I, Meyer J-N, Debus L, Pitra C, Ludwig A (2000). Evidence of mitochondrial DNA clones of Siberian sturgeon, *Acipenser baerii*, within Russian sturgeon, *Acipenser gueldenstaedtii*, caught in the River Volga.. Ecol Lett.

[28] Simons AM, Wood RM, Heath LS, Kuhajda BR, Mayden RL (2001). Phylogenetics of *Scaphirhynchus* based on mitochondrial DNA sequences.. Trans Am Fish Soc.

[29] Ruban GI, Kholodova MV, Kalmykov VA, Sorokin PA (2011). A review of the taxonomic status of the Persian sturgeon (*Acipenser persicus* Borodin).. J Appl Ichthyol.

[30] Timoshkina NN, Barmintseva AE, Usatov AV, Mugue NS (2009). Intraspecific genetic polymorphism of Russian Sturgeon *Acipenser gueldenstaedtii*.. Russ J Genet.

[31] Gessner J, Wirth M, Kirschbaum F, Krüger A, Patriche N (2002). Caviar composition in wild and cultured sturgeons – impact of food sources on fatty acid composition and contaminant load.. J Appl Ichthyol.

[32] Wuertz S, Groper B, Gessner J, Kruger T, Luckas B (2009). Identification of caviar from increasing global aquaculture production – Dietary capric acid as a labelling tool for CITES implementation in caviar trade.. Aquaculture.

[33] IUCN (2010). IUCN Red List of Threatened Species. Version 2010.4.. http://www.iucnredlist.org.

[34] Doukakis P, Babcock E, Sharov A, Pikitch EK, Baimukanov M (2010). Management and recovery options for Ural River beluga sturgeon based upon the first quantitative assessment.. Conserv Biol.

[35] Birstein VJ, Doukakis P, DeSalle R (1999). Molecular phylogeny of Acipenserinae and black caviar species identification.. J Appl Ichthyol.

[36] Doukakis P, Hanner R, Shivji M, Bartholomew C, Chapman D (2011). Applying genetic techniques to study remote shark fisheries in northeastern Madagascar.. Mitochondrial DNA.

[37] Zhang Z, Schwartz S, Wagner L, Miller W (2000). A greedy algorithm for aligning DNA sequences.. J Comp Biol.

[38] Stamatakis A (2006). RAxML-VI-HPC: maximum likelihood-based phylogenetic analyses with thousands of taxa and mixed models.. Bioinformatics.

[39] Stamatakis A, Ott M (2008). Efficient computation of the phylogenetic likelihood function on multi-gene alignments and multi-core architectures.. Phil Trans R Soc B.

[40] Lanave C, Preparata G, Saccone C, Serio G (1984). A new method for calculating evolutionary substitution rates.. J Mol Evol.

[41] Tavaré S, Some Mathematical Questions in Biology – DNA Sequence Analysis. (Miura RM (1986). Some probabilistic and statistical problems in the analysis of DNA sequences..

[42] Yang Z (1994). Maximum likelihood phylogenetic estimation from DNA sequences with variable rates over sites: approximate methods.. J. Mol Evol.

[43] Stamatakis A, Hoover P, Rougemont J (2008). A rapid bootstrap algorithm for the RAxML Web servers.. Syst Biol.

